# Dr. William J. Deane

**Published:** 1903-02

**Authors:** 


Dr. William J. Deane, U. of B. '95, of Buffalo, died at Kingston, Ont., from tuberculosis, December 31, 1902, aged 30 years.

